# Development of a Modified Version of the Spinal Function Sort (M-SFS): A Mixed Method Approach

**DOI:** 10.1007/s10926-015-9611-4

**Published:** 2015-10-23

**Authors:** Svenja Janssen, Maurizio A. Trippolini, Roger Hilfiker, Peter Oesch

**Affiliations:** 1 Department of Work Rehabilitation, Rehaklinik Bellikon, Suva Care, 5454 Bellikon, Switzerland; 2 School of Health Sciences, HES-SO Valais-Wallis, University of Applied Sciences and Arts Western Switzerland Valais, Sion, Switzerland; 3 Department of Research, Rehabilitation Centre Valens, Valens, Switzerland

**Keywords:** Back pain, Self-efficacy beliefs, Questionnaire, Work

## Abstract

*Purpose* To develop a modified version of the spinal function sort (M-SFS) by measuring work-related self-efficacy beliefs in patients with chronic low back pain. *Methods* A mixed method design consisting of three different methods (M1–3) was performed. In semi-structured interviews participants were asked how often they performed the activities of the 50 SFS items in 1 week, and which spinal postures and movements were associated with their back pain (M1). Quantitative analysis of previously obtained SFS data investigated internal consistency, unidimensionality, item response, and floor and ceiling effect (M2). Experts rated the SFS items based on their relevance (M3). The findings from these methods were used within a final scoring system for item reduction. *Results* From semi-structured interviews with 17 participants, eight new items emerged (M1). Quantitative analysis of 565 data sets (M2) revealed very high internal consistency of all items (Cronbach’s alpha = 0.98) indicating item redundancy; unidimensionality of the SFS was supported by principal component analysis; good item response was confirmed by Rasch analysis; and a floor effect of four items depicting very heavy material handling was found. Experts agreed on 8 out of the 50 SFS as relevant (M3). From the original SFS, 12 items met the predefined summary score of 9. *Conclusions* A modified version of the SFS with 20 items has been developed. Feasibility, reliability and validity of this modified version must be tested before it can be used in clinical practice.

## Introduction

Low back pain (LBP) and its consequences for society remains a global health problem [1–3]. However, less than 15 % of LBP can be explained by specific back diseases such as vertebral fracture, tumor, infection, inflammatory diseases, nerve root compression, spondylolisthesis, spinal stenosis and definite instability [4]. Nonspecific LBP (NSLBP) is not attributed to the above-mentioned specific causes and about 10 % of these patients develop chronic NSLBP [4].

The primary treatment goal in patients with chronic NSLBP is a return to work (RTW). The assessment of risk factors for non-return to work (N-RTW) plays an important role in their management [5]. Perceived self-efficacy is a relevant psychosocial factor contributing to the outcome in patients with chronic musculoskeletal pain [6]. According to Bandura, perceived self-efficacy affects how people behave in difficult situations, and people who doubt their capabilities shy away from tasks which they view as personal threats [7, 8]. Within the bio-psychosocial model of health it is suggested that work-related self-efficacy beliefs are more closely related to work disability than actual physical ability [9–12]. Consequently, it is recommended that the self-efficacy beliefs of patients with chronic NSLBP are measured, for example, by questionnaire [3].

Although questionnaires usually have many advantages, such as being cheap, easy to administer and to interpret, they also have limitations. The use of questionnaires depends on literacy and linguistic skills. These skills may be limited in patients with different mother tongues, resulting in lower response to questionnaires [13]. A way to overcome these limitations is through the use of picture-based questionnaires [14]. One questionnaire for the measurement of work-related self-efficacy beliefs is the spinal function sort (SFS) [15]. The SFS consists of 50 depicted items that are linked to demonstrable, specific work-related tasks that involve the spine. The respondent to the SFS is asked to rate the 50 various activities involving the spine on a 5-point scale from 1 (“able”) through 2, 3, and 4 (“restricted”) to 5 (“unable”) or, as a sixth possibility, to tick the question mark [“?” (don’t know)] if he or she is not sure of being able to perform the activity. A maximal point score of 200 can be reached. The SFS has been translated and validated in different languages and is used in several countries [16–19]. It is used in work rehabilitation programs in conjunction with functional capacity evaluation (FCE) to compare work-related self-efficacy beliefs with observed functional capacity [19–21].

Several studies have investigated the measurement properties of the SFS [16–18, 22] revealing adequate reliability, construct, and predictive validity for RTW at 1-year follow up for patients with long-term work disability due to chronic NSLPB [17]. Furthermore, high internal consistency, Cronbach’s alpha >0.95, indicating item redundancy, is reported [17, 18, 22]. In addition, four items showed floor effects with >85 % of the participants, who perceived themselves as unable to perform the displayed activity [17, 18]. Two studies concluded that the SFS could be improved by item reduction as well as by updating some of the old-fashioned pictures [17, 18]. Furthermore, the current version of the SFS does not include items that describe prolonged work postures, such as sitting or standing [23], which are reported as risk factors for LBP [24, 25]. Based on these findings, the purpose of this study was to develop a modified version of the SFS (M-SFS) for patients with chronic NSLBP.

## Methods and Material

### Study Design

**Fig. 1:**
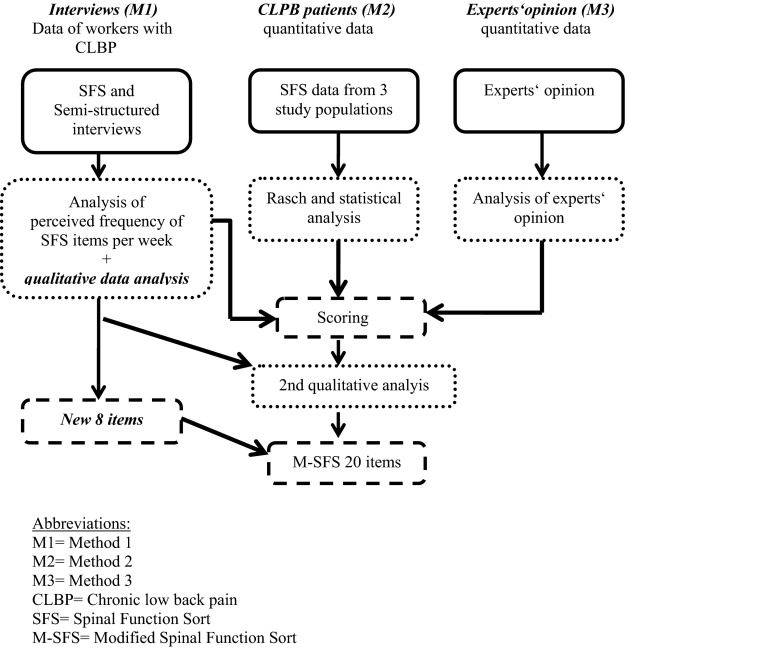
Study design

A mixed methods design was used in this study to modify the SFS [26, 27]. Mixed methods studies combine quantitative and qualitative methods to allow for a better understanding of the research problem instead of using one method alone [26]. The following three methods (M) were applied (see also Fig. 1):

- M1: Interviews with subjects with CLBP
- M2: Quantitative analysis of the SFS items based on data from previous studies [17, 21, 28]
- M3: Experts’ opinions.

### M1: Interviews

#### Participants

Subjects were recruited from the personnel (health care professionals, technicians, office workers, etc.) of the rehabilitation center at Bellikon, Switzerland. Inclusion criteria were: nonspecific CLBP for more than 3 months, aged between 18 and 65 years, no other severe disease or permanent injuries of the spinal cord, sufficient German language ability to be able to answer questions in the interviews, and a completed written informed consent form. Pregnancy was an exclusion criterion for female participants.

Ethics approval for this mixed methods study design was granted from the Medical Ethics Committee of the Canton Aargau, Switzerland (EK: 2012/073). All participants signed a written informed consent form.

#### Frequency Analysis

The interview consisted of three parts. Participants first completed the SFS plus an adapted version of the SFS asking for the perceived weekly frequency (“often,” “sometimes,” “seldom,” “never”) of each task depicted in the SFS. Completion of the questionnaires took 15–20 min in total. The perceived frequency of these tasks was viewed as an indication from the patient’s perspective, of the relevance of these items in everyday life.

#### First Qualitative Analysis

Semi-structured interviews with the participants were then conducted asking which spinal postures and movements (that are not included in the SFS) they believed to be associated with their back pain. The results of the interviews should help to integrate the patients’ perspective on postures and activities and their association with back pain into the modified version of the SFS. Participants were asked two phenomenological research questions which were used to lead the qualitative analysis process for exploring the themes of the new postural tolerance items [29]. The two research questions were: “During which activities do you feel restricted because of your back pain? Are they mentioned in the questionnaire?” (German: *Gibt es Tätigkeiten, die Ihnen Schwierigkeiten aufgrund Ihrer Rückenbeschwerden bereiten? Welche sind das und sind diese im Fragebogen benannt?*), and “Which prolonged postures cause pain in your back?” (German: *Welche Positionen die Sie über längere Zeit einnehmen lösen Schmerzen in Ihrem Rücken aus?*).

#### Second Qualitative Analysis

Finally, participants were asked which SFS items they experienced as redundant. Those items were excluded from the final M-SFS.

#### Interview Process

Participants had the opportunity to speak out loud throughout the whole of the interview process. This method of the three-part test is recommended for the qualitative examination of questionnaires [30]. The duration of the interview was 40–50 min per participant, including filling out the questionnaires.

The interviews were conducted by the first author. All interviews were audio-recorded and transcribed verbatim by a secretary who was not involved in the analysis process. The transcribed interviews were analyzed using the software ATLAS.ti for qualitative data analysis and themes were evaluated with the method of meaning units [31]. The sample was considered as saturated when no new themes emerged from the interviews [29, 32].

#### Measures of Validity

The interviewer has been working for 4 years as a physiotherapist in work-related rehabilitation and has regularly treated patients with chronic NSLBP for 6 years. The research and survey questions were discussed and selected by the study team before the interviews began and three pilot interviews were conducted. After the pilot interviews the formulation of phrases was adapted.

After every interview, an interview report was drawn up by the interviewer. As a quality check, two of the interviews were additionally transcribed by the interviewer and the texts compared with the secretary’s transcription. The analysis of the interview data was verified by the second author (also a physiotherapist), who did not take part in the interviews. Disagreement on the analysis and results was discussed.

To avoid the risk of observer bias by the interviewer, two randomly selected interviews were video-recorded. Two experienced psychologists independently checked both videos. This provided the opportunity to analyze the data from a different perspective and to check for suggestive questions. The psychologists found no signs of suggestive questioning.

### M2: Quantitative Analysis of Previous SFS Data

#### Participants

For the quantitative analysis, data were used from three previously published studies, one RCT and cross sectional studies [17, 21, 28]. Patients were participating in a function-oriented work rehabilitation program. Patients from two studies, who were referred to the Valens rehabilitation center, were aged between 20 and 55 years, suffered from chronic NSLBP (mean duration 1154 days), had no acute secondary diseases (e.g., vertebral fracture, tumor, infection, inflammatory diseases, nerve root compression and others), and had been on sick leave for a minimum of 6 weeks during the previous 6 months [17, 21]. The third study included patients who were referred to the rehabilitation center at Bellikon, who were no older than 60 years, and had suffered from persistent pain after a traumatic accident (referred to rehabilitation more than 9 months (median) after the accident) without acute secondary diseases or permanent injuries [28]. All patients gave written informed consent as requested by the local Medical Ethics Committee.

#### Statistical Analysis

Unless reported otherwise, all statistical analyses were performed with SPSS (Statistical Package for Social Sciences, Version 21, IBM Corp.).

#### Internal Consistency

Internal consistency was calculated by item-to-total correlations and Cronbach’s alpha. Analysis was also performed with half of the items, forming two groups (one with the even numbered items and one with the odd numbered items). Optimal consistency for measurements at group level was considered when alpha value was between 0.7 and 0.9. Values <0.7 may be indicative for items measuring different traits, values >0.9 may be indicative for item redundancy [33].

#### Unidimensionality

Unidimensionality of the 50 SFS items was evaluated using principal component analysis (PCA) with Kaiser normalization and varimax rotation using the software R [34]. An eigenvalue criterion of 1.0 was used for the factor analysis.

#### Item Response

The fit of the items to the Rasch model was examined with mean square infit and outfit statistics from Rasch analysis [35]. We interpreted values between 0.5 and <1.5 as a good fit, low but still sufficient if the item fit was between 1.5 and 2, and insufficient fit was defined as values above 2 or below 0.5 [36].

#### Floor and Ceiling Effect

Floor or ceiling effect was set if an item was scored >85 % at the lowest or highest score of the 6-point scale of the SFS [37].

### M3: Experts’ Opinions

Four experts (the authors), with more than 5 years’ experience as physiotherapists in work-related rehabilitation settings, were requested to independently score which of the 50 SFS items were important in relation to patients with chronic NSLBP. Every expert rated the relevance of every SFS item with “yes” or “no” (y/n). All four ratings were matched together and points were given for each item within the scoring.

### Final Item Selection for the M-SFS

Final item selection was performed by applying the adapted Stanton criteria for length reduction of self-reporting scales [38]. For item selection, a summary scoring was developed for each SFS item based on the results of methods 1–3. The summary score consisted of the following criteria: perceived frequency of each item during a common week, item-to-total correlation, principal component analysis, Rasch analysis, floor and ceiling effect and experts’ opinions on the relevance of the items (see Table 1). Items below the total summary score of 9, from a maximal 12 points, were not selected for the SFS shortened questionnaire. The selected items from the summary scoring and the new themes mentioned in the interviews, created the new questionnaire.

**Table 1:** Scoring for final item selection

Category	Cut-off	Scores
Perceived weekly frequency of activities (“often” and “sometimes”)	>13 times	2
	6–12 times	1
	<6 times	0
Item-to-total correlation	0.6–0.9	2
	0.4–0.59	1
	<0.4 or >0.9	0
Principal component analysis	Loading on one factor >0.5	2
	Loading on two or no factor >0.5	0
Rasch analysis (item fit)	0.5–1.5	2
	>1.5 to 2	1
	>2 or <0.5	0
Floor or ceiling effect	<85 %	2
	>85 %	0
Experts’ opinion on relevance of SFS item (“yes”)	All experts	1
	3 out of 4	0.75
	2 out of 4	0.5
	1 out of 4	0.25
	None	0

*SFS* spinal function sort

## Results

### M1: Interviews

#### Participants

Seventeen workers with chronic NSLBP from several professions at the rehabilitation center in Bellikon (Aargau), Switzerland, participated in the interviews. Eight men and nine women with a mean age of 44 years were interviewed. Reported current pain was mentioned with 1.6 in mean, and standard deviation (SD) of 1.4 on a numeric rating scale (NRS 0–10). The average duration of pain in days was 3796. Of the sample, 35 % had more than 9 years of school education and were married. Single marital status was reported by 47 %. German was spoken by 82, and 88 % were in possession of a work contract with a position as worker or office worker in 65 % of all mentioned work roles (see Table 2).

**Table 2:** Demographics of the interviewed participants with chronic NSLBP (n = 17)

Variable	n	Mean	SD	%
Age	17	44	12	
Self-reported pain (NRS)	17	2	1	
Duration of pain (days)	17	3796	4554	
Gender				
Male	8			47
Female	9			53
Education (years at school)				
6 years	1			6
7–9 years	10			59
>9 years	6			35
Marital status				
Single	8			47
Married	6			35
Divorced/parted	2			12
Unknown	1			6
Native language				
German	14			82
Italian	1			6
Spanish	1			6
Turkish	1			6
Work-contract				
Yes	15			88
No	2			12
Work-position				
Worker/office worker	11			65
Superior/team leader	5			29
Cadres/manager	1			6

*NRS* numeric rating scale, min. 0–max. 10

*NSLBP* nonspecific low back pain

#### Frequency Analysis

The analysis of the frequency of performance of the SFS items per week showed that the following six items were rated as activities performed on a daily basis (i.e., often): bending (items 1 and 2), lifting 10 kilos (item 15), forward standing for more than 5 min (item 19), and trunk rotation (items 30 and 32). Fifteen items were assessed as tasks that were performed three to four times each week (i.e., sometimes).

#### First Qualitative Analysis

In total, there were 20 subjects available for data collection but three subjects were excluded from the study: two subjects did not agree to be audio-recorded and one subject had filled out the SFS on many previous occasions. In the latter case, this subject was not interviewed to avoid the potential influence of this specific knowledge on the research questions. After 17 interviews no new themes emerged and, therefore, saturation was reached [29, 32]. Several themes emerged following the thematic analysis of the transcribed interviews. A total of eight themes of postures were mentioned: prolonged sitting, standing, walking, forward standing, crouching, forward sitting, bending, and whole body vibration (see Table 3). Examples of these themes are:

**Table 3:** Summary of the answers to the research question collected during the semi-structured interviews

Research question: Which prolonged posture causes low back pain for you?			
Quote	Meaning unit	Consent meaning unit	Code
*Quotes for prolonged walking*			
(Are there other prolonged postures which cause you back pain? Except mentioned long walking?)	Prolonged back pain causing postures, except long walking?	Prolonged postures, except long walking?	Prolonged walking
During walking and sitting. Ok, now I have pain, too. (And more if you are sitting over a short or a long time?) Sitting over a long time	During walking and sitting over long time	Walking and sitting over long time	Prolonged walking and sitting
*Quotes for prolonged forward standing*			
Bent forward. Well, doing something in this posture. (So, prolonged forward standing?) Exactly	So, prolonged forward standing? Exactly	Prolonged forward standing	Prolonged forward standing
Forward bent standing, yes	Forward bent standing	Forward bent standing	Prolonged forward standing
*Quote for prolonged forward sitting*			
… if I am sitting … like in a forward bent position. During sitting	Sitting in a forward bent position	Sitting forward	Prolonged forward sitting
*Quote for whole body vibration*			
(Any other activities where you have pain in your back? Something in your leisure time or at work?) During sledging	(Something in your leisure time or at work?) During sledding	During sledding	Whole body vibration
*Quote for bending*			
So, e.g., you have to tie the iron, then you are bent most of the time, except it is not a wall, if it is a ceiling or a floor you are often bending	Then you are bending most of the time, except it is not a wall, if it is a ceiling or a floor you are often bending	You are often bending	Often bending
Above all things bending	Bending	Bending	Bending
Forward bent postures over time That’s difficult, too	Forward bent postures over time	Prolonged bent postures	Long bent postures
… in bent posture	Bent posture	Bent posture	Bent posture
*Quotes for prolonged crouching*			
Or, well, going on your knees or cowering for a long time, this could not be sustained	To be on your knees or cowering for a long time	Cowering over a long time	Long cowering
… and even longer prolonged cowering, too If you just go down for a short time, it’s not like that bad …	And even longer prolonged cowering too	Longer prolonged cowering	Long cowering
Long forced positions, I would call it	Long forced posture	Long cowering posture	Long cowering
*Quotes for prolonged standing*			
Yes, standing still	Standing calm over time	Standing over time	Long standing
Yes, standing on a ladder	Standing calm over time	Standing over time	Long standing
Standing calm, queuing somewhere	Standing calm over time	Standing over time	Long standing
(Would you see forward bent sitting and standing in prolonged postures as a problem for you?) Yes. Just catching something and putting it away is not a problem, but holding this position	Long forward bent sitting and standing	Long forward bent sitting and standing	Long sitting and standing
*Quotes for prolonged sitting*			
Long sitting during driving a car, for example	Long sitting during driving a car	Long sitting	Long sitting
It hurts, when I’m sitting. So I prefer standing	It hurts, when I’m sitting	Long sitting	Long sitting
… long sitting	Long sitting	Long sitting	Long sitting
Well now, sitting without a back rest. A chair, e.g., if I’m sitting too long, it hurts. Then I have to stand up and relax for a time, always	Long sitting without back rest. Then I have to stand up and relax for a time, always	Long sitting without back rest	Long sitting
… with the problem of intervertebral disc of the lumbar column, there it was long sitting of course	There it was long sitting	It was long sitting	Long sitting
… well, after which postures does the pain come but, e.g., longer train trips or generally sitting, it can be in a car, for over 2 or 2.5 h	Longer train trips, generally sitting, in a car, over 2 or 2.5 h	Generally sitting over 2 or 2.5 h	Long sitting
(Would you see forward bended sitting and standing in prolonged postures as a problem for you?) Yes. Just catching something and putting it away is not a problem, but holding this position	Long forward bending sitting and standing	Long forward bending sitting and standing	Long sitting and standing
Long sitting, I think because I was sitting over a long time	Because I was sitting over a long time	Sitting over a long time	Long sitting
So, if I have a prolonged posture, which causes me pain? Except sitting, rather nothing else, no	Except long sitting, rather nothing else, no	Except long sitting, nothing	Long sitting
During walking and sitting. Ok, now I have pain, too. (And more if you are sitting over a short or a long time?) Sitting over a long time	During walking and sitting over long time	Walking and sitting over a long time	Long walking and sitting

1. theme of prolonged sitting: (quotation) “Long sitting during driving a car, for example”.
2. theme of prolonged standing: (quotation) “Standing calm, queuing, somewhere”.
3. theme of prolonged walking: (quotation) “During walking …”.
4. theme of prolonged forward standing: (quotation) “Bent forward”.
5. theme of prolonged crouching: (quotation) “Kneeling or cowering for a prolonged time”.
6. theme of prolonged forward sitting: (quotation) “… if I am sitting … like in a forward bent position”.
7. theme of prolonged bending: (quotation) “Bending over something …”.
8. theme of whole body vibration: (quotation) “During sledging, bus driving”.

#### Second Qualitative Analysis

During the interviews participants reported that five out of the 17 items—i.e., lift 10 pounds from floor (item 10), unload 20 pounds (item 13), lift a 20-pound tool box from floor (item 16), get out of an automobile (item 32) and sweep with a broom (item 40)—were redundant. Hence, these five items were excluded to further reduce the number of items.

### M2: Quantitative Analysis of Previous SFS Data

#### Participants

A total of 565 patients were previously investigated [17, 21, 28]. The proportion of males was 54 %, mean age was 43 years, and mean duration of LBP was 1154 days. All demographic characteristics are shown in Table 4.

**Table 4:** Demographic characteristics of patients with chronic NSLBP (n = 565)

Variable	n	Mean	SD	%
Age	565	43	10.2	
Self-reported pain (NRS)	561	5	1.8	
Duration of pain (days)	541	1154	2011.8	
Gender				
Male	303			54
Female	262			46
Education (years at school)				
6 years	256			46
7–9 years	276			50
>9 years	25			4
Marital status				
Single	111			20
Married	173			66
Divorced/parted	75			13
Unknown	5			1
Native language				
(Swiss) German	307			54
French	7			1
Italian	21			4
Spanish	37			6
Portuguese	14			2
Serbo-Croatian	90			16
Albanian	20			3
Turkish	15			3
Other^a^	54			10
Work-contract				
Yes	274			49
Do not know	89			16
No	181			32
Other^b^	14			2
Work-position				
Unskilled worker/trainee	182			32
Worker/office worker	325			57
Superior/team leader	24			4
Cadres/manager	6			1
Owner/self-employed	24			4
Unknown	3			0.5

*NRS* numeric rating scale min. 0–max. 10

^a^Native language: other (English, Croatian, Macedonian, Slovenian, Bosnian)

^b^Work status: other (non-retired, temporary employment, uncertain)

#### Internal Consistency

Item-to-total analysis for all items showed a value of >0.6. Cronbach’s alpha value for all items was 0.98. When half of the items—one group with even and one group with odd numbered items—were analyzed, Cronbach’s alpha value for both groups was 0.96. Four items (items 45–48) asking about heavy material handling tasks with 50 kg had item-to-total correlation values of <0.40 (item 45: 0.36, item 46: 0.39, item 47: 0.33, item 48: 0.36).

#### Unidimensionality

The principal component analysis (PCA) revealed six components with eigenvalues exceeding 1. Items 45, 47, and 48 all loaded with high values over 0.9 on the second, and item 46 on the fourth factor. Six items loaded on any factor of the six components, that is, items 4 (pushing and pulling), 22 (crouching), 33 (carrying 5 kg), 34 (carrying 15 kg), 37 (climbing a ladder), and 38 (climbing a ladder with 10 kg).

#### Item Response

The infit and outfit mean square fit values from the Rasch analysis were consistently between 0.5 and 1.5 for all items, except for items 45–48, with values of >2.0.

#### Floor and Ceiling Effect

Four items (45–48), that is, tasks with lifting 50 kg, showed a floor effect of >85 % of the included subjects.

### M3: Experts’ Opinions

Eight out of the 50 SFS items were consistently rated by all experts as important in relation to patients with chronic NSLBP (item 8: lower 5 kg from a bench to a floor, item 11: lifting 10 kg into trunk of an automobile, item 16: lifting 10 kg from the floor to a bench, item 19: wash dishes at a sink, item 27: load or unload dishwasher, item 34: carrying 15 kg over 15 m, item 35: carrying 10 kg over 30 m, and item 44: lifting 25 kg from the floor to a bench).

### Final Item Selection: Scoring

For M-SFS, eight new prolonged body postures, which were claimed by the interviewed participants to cause LBP, were chosen from the 17 semi-structured interviews. Of the existing 50 items, 17 scored more than 9 points and were selected based on six criteria from the mixed methods approach consisting of M1, 2, and 3. After the second qualitative analysis, 12 items remained for the M-SFS. Six of these 12 items (items 8, 9, 11, 12, 15, and 50) in the new questionnaire describe tasks of lifting weights from 2.5 to 15 kg. Activities where the spine is in a forward bent position are depicted in two items (items 3 and 19). Carrying weights of 10 kg over a distance of 30 m is represented in one item (item 36). One item requires rotation and lateral flexion of the spine (item 30). Repetitive bending and rising of the trunk with very low weight is referred to in one item (item 27) and another asks for lifting of 25 kg (item 44). This results in 20 items represented in the M-SFS (see “Appendix”).

## Discussion

A modified version of the SFS was achieved using a mixed methods approach resulting in a total of 20 items [39]. Semi-structured interviews with patients with chronic NSLBP revealed eight prolonged postures or movements that were associated with their LBP. These are: sitting, standing, walking, forward standing, crouching, forward sitting, whole body vibration, and repetitive bending. From the original 50 items of the SFS, 12 remained after item selection according to the adapted Stanton criteria for length reduction of self-reporting scales [38]. All items depicting lifting tasks with weights over 25 kg (items 45–48) were excluded from the M-SFS by the applied scoring system. This is in line with the current European work safety guidelines which no longer recommend lifting tasks with weights over 25 kg [18].

The most relevant findings from the quantitative analysis of 565 SFS’s obtained in previous studies [17, 21, 28] were the high Cronbach’s alpha values of 0.98 of all items, and 0.96 if the sample was split into two groups—one group with even and one group with odd numbered items; six items loaded on any of the with PCA identified six components of the SFS; mean square fit values from the Rasch analysis between 0.5 and 1.5 for all items except for items 45–48 with values >2.0; and large floor effects of four items depicting lifting tasks of 50 kg (items 45–48). These findings strongly support item reduction and modification of the SFS, as suggested in previous studies [17, 18, 40].

The strength of this study is the mixed methods approach which combined interviews with subjects with CLBP, a quantitative analysis of the SFS items based on a high number of datasets obtained in previous studies [17, 21, 28], and experts’ opinions. The findings from these methods were used within the final scoring system as recommended by Stanton et al. [38] for item reduction of self-reporting scales. An arbitrary cut-off value of 9 points was determined for this study. An item could have reached a maximal 12 points in the scoring, so it was supposed that an absolute majority over 75 % of the maximum score would be adequate for a cut-off value. A further strength is the use of semi-structured interviews that allowed for new items of LBP-causing postures to be explored.

A weakness of this study is the small number of experts. Only four experts rated the relevance of the SFS items using a dichotomous questionnaire. Furthermore, the sample of participants included in the quantitative and qualitative analysis consisted of patients with chronic NSLBP. Therefore, the findings of this study may not be appropriate for patients with acute LBP or patients with other disorders.

It might also be argued that the M-SFS is lacking the internal validity check that was previously used. This aimed at identifying inconsistencies in answers and was achieved by including two identical items. However, we performed a post hoc analysis with the 565 SFS’s obtained in previous studies revealing that inconsistencies between these identical items were extremely rare. We therefore feel that such a check is redundant. However, further research must be performed to clarify whether the internal validity check is indeed redundant. Further research must also investigate the feasibility, test–retest reliability, and construct validity of the M-SFS. A study with patients with chronic NSLBP is planned to investigate these measurement properties.

## Conclusion

Based on the results of a mixed methods approach, a modified SFS requiring less administration time was developed. This consists of 12 items from the existing SFS and eight new items that include patient’s beliefs about back pain causing postures and movements. Feasibility, reliability, and validity of the M-SFS need to be explored in future studies before it can be used in clinical practice.

## Appendix

See Table 5.

**Table 5:** Modified spinal function sort (M-SFS)

		Able	Restricted			Unable
1	Place or retrieve a 2.5 kg can between waist and overhead	1	2	3	4	5
2	Lower a 10 pound milk crate from a bench to the floor	1	2	3	4	5
3	Lift a 5 kg milk crate from the floor to eye level	1	2	3	4	5
4	Load a 10 kg grocery bag into the trunk of an automobile	1	2	3	4	5
5	Lower a 10 kg milk crate from eye level to the floor	1	2	3	4	5
6	Unload two 5 kg grocery bags from the trunk of an automobile	1	2	3	4	5
7	Carry two 5 kg sacks of groceries for 30 m	1	2	3	4	5
8	Lift a 25 kg tool box from the floor to a bench	1	2	3	4	5
9	Wash dishes at a sink	1	2	3	4	5
10	Load or unload a dishwasher	1	2	3	4	5
11	Push and pull a vacuum cleaner	1	2	3	4	5
12	Get into an automobile driver’s seat	1	2	3	4	5
13	Stand for a prolonged time	1	2	3	4	5
14	Walk for a prolonged time	1	2	3	4	5
15	Stand bent forward over for a prolonged time	1	2	3	4	5
16	Crouch for a prolonged time	1	2	3	4	5
17	Sit bent forward for a prolonged time	1	2	3	4	5
18	Bend forward repeatedly	1	2	3	4	5
19	Sit on a chair for a prolonged time	1	2	3	4	5
20	Sit with whole body vibration for a prolonged time; e.g. a bus journey	1	2	3	4	5
